# Postoperative detection of *Klebsiella pneumoniae* in clinical cultures and recurrent primary sclerosing cholangitis after liver transplantation: a single-center retrospective cohort study

**DOI:** 10.3389/ti.2026.16698

**Published:** 2026-06-22

**Authors:** Jun Ichikawa, Takashi Ito, Katsunori Sakamoto, Shinya Okumura, Daisuke Ueda, Tokuyuki Yamashita, Hiroki Aoyama, Ryo Ataka, Satoshi Ogiso, Kazuyuki Nagai, Yoichiro Uchida, Takamichi Ishii, Etsuro Hatano

**Affiliations:** Department of Surgery, Graduate School of Medicine, Kyoto University, Kyoto, Japan

**Keywords:** clinical cultures, *Klebsiella pneumoniae*, liver transplantation, primary sclerosing cholangitis, recurrence of primary sclerosing cholangitis

## Abstract

Recurrent primary sclerosing cholangitis (rPSC) remains a significant challenge. Although *Klebsiella pneumoniae* (Kp) has been implicated in PSC pathogenesis, its association with rPSC remains unclear. We retrospectively analyzed 62 patients who underwent LT for PSC at our institution between 1996 and July 2024 and survived for more than 1 year. We assessed the association between Kp detection in postoperative clinical cultures and rPSC. rPSC was observed in 22 of 62 patients (35.5%). Kp was detected more frequently in the rPSC group than in the non-rPSC group (31.8% vs. 7.5%). In multivariate Cox regression analysis, Kp detection in clinical cultures (Hazard ratio: 3.83, 95% confidence interval: 1.44–10.21, p = 0.007) and identification of three or more bacterial species in clinical cultures (Hazard ratio: 0.31, 95% confidence interval: 0.12–0.78, p = 0.01) were indpendently associated with rPSC. Recurrence-free survival was significantly shorter in patients with Kp detection (10-year recurrence-free survival: 28% with Kp vs. 56.7% without Kp, p = 0.03). These findings suggest a potential association between postoperative Kp detection and rPSC.

## Graphical Abstract

**Figure:**
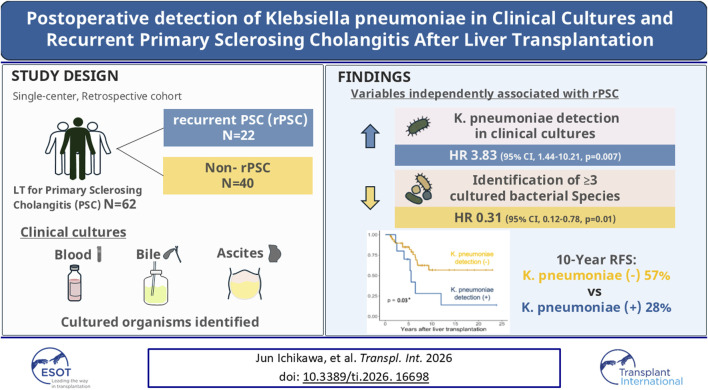

## Introduction

Primary sclerosing cholangitis (PSC) is a progressive cholestatic disease characterized by chronic inflammation and fibrosis of the intrahepatic and extrahepatic bile ducts, ultimately leading to cirrhosis and liver failure. Currently, there is no effective medical therapy, and liver transplantation (LT) remains the only curative treatment option [1].

Recurrence of the primary disease, termed recurrent PSC (rPSC), may occur after LT and is associated with impaired graft function and adverse long-term outcomes [1]. Various risk factors for rPSC have been reported, including active inflammatory bowel disease [2, 3], reduction of immunosuppressive therapy [4], older donor age [4, 5], a high preoperative Model for End-Stage Liver Disease (MELD) score [5], postoperative biliary complications [6], living donation from a first-degree relative [7], and cytomegalovirus (CMV) disease within 3 months post transplantation [7]. However, the reproducibility of these findings and the underlying mechanisms have yet to be clarified.

In recent years, increasing attention has been paid to the role of gut microbiota, particularly *Klebsiella pneumoniae* (Kp), in the pathogenesis of PSC [8]. Nakamoto et al. [8] reported that in a PSC mouse model, Kp disrupts the intestinal barrier, triggers immune responses via Th17 cells, and contribute to hepatic fibrosis. Hole et al. [9] analyzed colonic mucosal biopsy specimens from rPSC patients and demonstrated that these patients exhibited significantly greater dysbiosis compared with non-recurrent patients. They also reported that, although the difference was not significant, the abundance of *Klebsiella* genus was higher in rPSC patients than in non-recurrent patients. As Hole et al. [9] investigated *Klebsiella* at the genus level, the relationship between Kp and rPSC remains to be elucidated.

The aim of the present study was to investigate the association between Kp detection in postoperative clinical cultures and rPSC after LT.

## Patients and methods

### Patients and data collection

This study included 76 patients who underwent their first LT for PSC at a tertiary referral center between January 1996 and July 2024. For the analysis of rPSC, 12 patients who died within 1 year post-transplant and two recurrent cases for whom sufficient clinical information could not be obtained were excluded. Ultimately, 62 patients were enrolled in the final analysis. The study protocol was approved by the institutional ethics committee (No. R1473-8) and performed in compliance with the Helsinki Declaration.

Demographic characteristics, perioperative findings, and long-term outcomes were retrospectively collected from medical records. In addition to risk factors for rPSC, we collected microbiological and treatment-related variables, including the number of submitted culture tests, the types of organisms detected, the number of positive detections, and antimicrobial use. Data on first-time liver transplantation cases were included in the analysis.

### Definition of rPSC, assessment of Kp

rPSC was defined according to the criteria proposed by Graziadei et al [10]. Specifically, patients with a preoperative diagnosis of PSC were considered to have recurrence if, at 90 days or more after LT, they exhibited either multiple intrahepatic bile duct strictures on cholangiography or magnetic resonance cholangiopancreatography (MRCP), or histological findings consistent with fibrous cholangitis or fibro-obliterative lesions. Other potential causes, such as ischemic cholangitis, chronic ductopenic rejection, and anastomotic stricture within 90 days post-transplantation, were excluded.

For Kp detection in rPSC cases, Kp positivity was defined as those with Kp identified in blood, bile, or ascitic fluid cultures obtained between LT and rPSC diagnosis, whereas in non-rPSC cases, as those with Kp identified in same clinical cultures between LT and the end of follow-up. The same criteria were applied to the evaluation of other bacterial species and the number of clinical culture tests performed. Only clinical cultures with species-level identification were included; those identified only to the genus level were excluded.

### Operative procedure and management

All procedures and postoperative management followed the standard protocol of our institution in all cases. Roux-en-Y hepaticojejunostomy was the standard biliary reconstruction method for PSC cases. Biliary stents were placed intraoperatively and externally drained through the blind end of the duodenum. Patients were discharged with the stent in place, which was removed under fluoroscopic guidance after approximately 3 months if no complications occurred. Preoperative prophylactic antibiotics typically included ampicillin and second-generation cephalosporins. If preoperative clinical cultures were positive, antibiotic selection was adjusted based on sensitivity testing. Antibiotics were continued for 2 days after LT and discontinued if no signs of infection were observed. When infection was suspected, based on fever, elevated liver enzymes, or inflammatory markers, blood, bile, ascites, and urine cultures were obtained, and empirical antibiotics administered. In addition, clinical cultures were submitted at the surgeons’ discretion. The same protocol was applied in the case of readmission due to fever or similar symptoms. Bile samples were collected aseptically from a stent placed intraoperatively until its removal. Thereafter, further bile samples were collected via endoscopic retrograde cholangiopancreatography or percutaneous transhepatic cholangial drainage. Ascitic fluid was collected via intraperitoneal drains or paracentesis. Immunosuppressive regimens consisted of dual or triple therapy combining a calcineurin inhibitor with steroids, mycophenolate mofetil, and everolimus, which were administered from 1 month after transplantation. Tapering of immunosuppressive agents during the maintenance phase was performed at the surgeon’s discretion. Rituximab was administered as induction therapy for ABO-incompatible living donor liver transplantation (LDLT) or in patients with preoperatively strong positive donor-specific antibodies. Histological diagnoses of acute and chronic rejection were made according to the Banff criteria.

### Statistical analysis

Continuous variables were expressed as medians with interquartile ranges (IQR) and compared using the Mann–Whitney U test. Categorical variables were compared using the chi-square test or Fisher’s exact test. Kaplan–Meier method and log-rank test were used for survival analyses. Factors associated with rPSC were evaluated using a Cox proportional hazards model, with death or re-transplantation treated as censoring events. Cut off values for continuous variables were determined by receiver operating characteristic curve analysis and the Youden index or based on previously published studies and clinical relevance. Variables considered clinically and statistically relevant were included in the multivariable analysis, with model selection via the backward stepwise method. In addition, bootstrap resampling with 1,000 iterations was conducted to assess the robustness and stability of the multivariable model estimates. All statistical analyses were performed using R software (version 4.3.0).

## Results

### Liver transplantation outcomes for PSC at our institution

**FIGURE 1:**
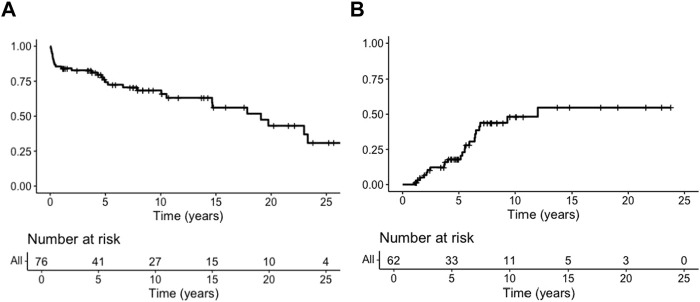
Patient and graft outcomes after liver transplantation for primary sclerosing cholangitis (PSC) (n = 76). **(A)** Overall patient survival. **(B)** Cumulative incidence of recurrent primary sclerosing cholangitis (rPSC).

Among 76 patients who underwent initial LT for PSC at our institution during the study period, the median age was 36.0 years (IQR, 25.8–49.0 years), 66 were adults (86.8%), and 36 were male (47.4%). LDLT was performed in 64 cases (84.2%), deceased donor liver transplantation in eight cases (10.5%), and domino transplantation in four cases (5.3%). ABO-incompatible LT was performed in eight cases (10.5%). The median follow-up period was 75.1 months (IQR, 37.9–168.0 months; mean, 105.1 months). rPSC occurred in 24 patients (31.6%), with a median time to recurrence of 55.0 months (IQR, 26.4–76.9 months). Kaplan–Meier curves for overall survival (OS) and recurrence rates among the 76 patients are shown in Figure 1. The 5-, 10-, and 15-year OS rates were 74.3%, 68.4%, and 56.1%, respectively (Figure 1A). The 5-, 10-, and 15-year recurrence rates were 20.9%, 50.1%, and 56.3%, respectively (Figure 1B). Primary graft survival (PGS) rates were 69.6% at 5 years, 50.1% at 10 years, and 25.0% at 15 years.

### Patient demographics

The background characteristics of the 62 patients included in the recurrence analysis are summarized in Table 1. rPSC occurred in 22 patients (35.5%), and 40 patients (64.5%) were in the non-recurrent group. The median age in the rPSC group was 37.0 years (IQR, 26.5–47.5), and 41.0 years (IQR, 27.5–50.0) in the non-rPSC group, with no significant difference. There were no significant differences between both groups in the proportion of adult cases, sex, preoperative MELD score, Mayo PSC risk score, Child–Pugh classification, history of ulcerative colitis (UC), or human leukocyte antigen (HLA)-DR15 positivity. Regarding donor factors, the median donor age was 48.0 years (IQR, 37.0–55.0) in the rPSC group and 41.0 years (IQR, 33.0–51.0) in the non-rPSC group (p = 0.12), with no significant difference. There were no significant differences between both groups in sex mismatch, ABO-incompatible transplantation, first-degree-related donors, or number of HLA mismatches. The presence of donor HLA-DR15 also did not significantly differ between the groups (rPSC vs. non-rPSC = 12 cases [57.1%] vs. 13 cases [35.1%], p = 0.18). Surgical factors, including LDLT, operative time, blood loss, cold ischemia time, warm ischemia time, and biliary reconstruction method, were not significantly different. Postoperative factors, including major complications, biliary complications, CMV infection, acute rejection, and immunosuppressive regimen at 1 year post-LT (alone/dual/triple), also showed no significant differences between the groups. On the other hand, active postoperative UC was significantly more frequent in the rPSC group (rPSC vs. non-rPSC = 10 cases [45.5%] vs. six cases [15.0%], p = 0.02). As for bacterial cultures, the median number of clinical cultures submitted postoperatively (including blood, bile, and ascitic fluid) was 26.5 (IQR: 11.3–46.3) in the rPSC group and 27.5 (IQR: 19.0–35.5) in the non-rPSC group, with no significant difference (p = 0.66). However, the detection rate of Kp was significantly higher in the rPSC group (seven cases [31.8%] vs. three cases [7.5%], p = 0.03). No significant differences were observed between the groups in the identification of three or more bacterial species or the use of antibiotics.

**TABLE 1:** Patient characteristics.

Variables	rPSC (n = 22)	Non-rPSC (n = 40)	p
Recipient factors			
Age (years old)	37.0 (26.5–47.5)	41.0 (27.5–50)	0.70
Adult/Pediatric	20/2	36/4	>0.99
Sex, male/Female	8/14	23/17	0.19
MELD score	18.5 (12.3–21.8)	15.0 (9.0–20.3)	0.26
Mayo PSC risk score	3.0 (2.1–3.4)	2.8 (1.9–3.6)	0.89
Child–Pugh class A/B/C	3/8/11	5/13/22	0.93
History of UC (yes/no)	8/14	19/21	0.56
HLA-DR15 (yes/no)	7/15	21/19	0.19
Donor factors			
Age (years old)	48.0 (37.0–55.0)	41.0 (33.0–51.0)	0.12
Sex, M/F	12/10	28/12	0.35
Sex mismatch (yes/no)	12/10	23/17	>0.99
ABO-incompatible (yes/no)	3/19	3/37	0.66
First degree relationship (yes/no)	14/8	24/16	>0.99
HLA mismatch number (≥4/<4)	16/5	31/6	0.72
HLA-DR15 (yes/no)	12/9	13/24	0.18
Operativeffactors			
LDLT/DDLT/Domino	18/3/1	32/5/3	0.90
Operation time (min)	685 (561–828)	715 (654–891)	0.29
Estimated blood loss (mL)	2,400 (1,430–4,980)	2,900 (2010–5,530)	0.67
Cold ischemic time (min)	90.0 (58.0–227)	87.0 (43.5–222)	0.61
Warm ischemic time (min)	40.0 (33.5–43.8)	36.0 (32.3–39.0)	0.30
Hepaticojejunostomy/duct-to-duct	1/25	1/38	>0.99
Postoperative factors			
Major complication (yes/no)	6/16	10/30	>0.99
Biliary tract complication (yes/no)	4/18	7/33	>0.99
CMV infection (yes/no)	8/14	20/20	0.44
Acute rejection (yes/no)	10/12	18/22	>0.99
Active UC after LT (yes/no)	10/12	6/34	0.02^*^
Immunosuppression 1 year after LT (single/dual/triple)	5/10/7	3/16/21	0.14
Cultures and antibiotics			
No. of total clinical cultures (blood, biliary juice, ascites)	26.5 (11.3–46.3)	27.5 (19–35.5)	0.66
≥3 bacterial species identified in clinical cultures (yes/no)	12/10	26/14	0.59
Kp (+) in clinical cultures after LT (yes/no)	7/15	3/37	0.03^*^
Oral antibiotic treatment with MNZ and VCM (yes/no)	5/17	5/35	0.31
Intravenous antibiotic therapy with MNZ, VCM, and TEIC (yes/no)	9/13	22/18	0.56

Continuous variables are described as median (interquartile range [IQR]). RPSC, Recurrent primary sclerosing cholangitis; MELD score, Model for end-stage liver disease score; UC, Ulcerative colitis; HLA, Human leukocyte antigen; LDLT, Living donor liver transplantation; DDLT, Deceased donor liver transplantation; CMV, Cytomegalovirus; LT, Liver transplantation; Kp, *Klebsiella pneumoniae*; MNZ, Metronidazole; VCM, Vancomycin; TEIC, Teicoplanin. *p values <0.05.

### RPSC impairs primary graft survival but not OS

**FIGURE 2:**
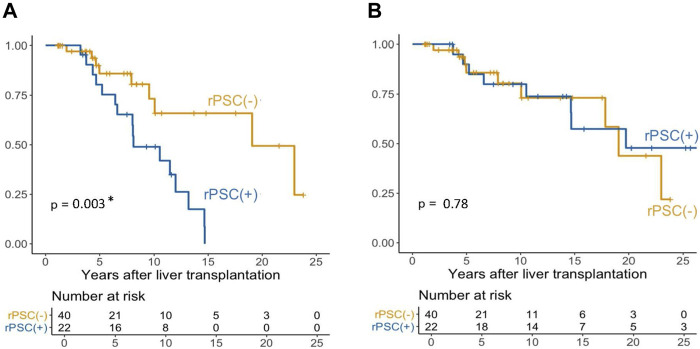
Patient and graft outcomes after liver transplantation for primary sclerosing cholangitis (PSC) (n = 62). **(A)** Graft survival stratified by the presence or absence of recurrent PSC (rPSC). **(B)** Overall patient survival stratified by the presence or absence of rPSC. *p values <0.05. RPSC: Recurrent primary sclerosing cholangitis.

Kaplan–Meier curves for PGS and OS stratified by recurrence status among the 62 patients are shown in Figure 2. The 5- and 10-year PGS rates in the rPSC group were 80.4% and 49.0%, respectively, with a median graft survival of 97.2 months. In the non-rPSC group, the 5- and 10-year PGS rates were 85.9% and 73.3%, respectively, with a median graft survival of 229.2 months. PGS was significantly lower in the rPSC group (p = 0.003, Figure 2A). OS stratified by recurrence status showed no significant difference between both groups: in the rPSC group, 5-, 10-, and 15-year OS rates were 90.0%, 80.0%, and 57.4%, respectively, with a median survival of 236.4 months; in the non-rPSC group, the corresponding rates were 85.8%, 80.4%, and 73.1%, with a median survival of 229.2 months (p = 0.78, Figure 2B).

### Kp detection and lower diversity of cultured organisms associated with rPSC

Table 2 summarizes the results of univariate and multivariate Cox proportional hazards models assessing variables associated with rPSC. In the univariate analysis, postoperative active UC (p = 0.03), Kp detection (p = 0.03) and the identification of three or more bacterial species in clinical (p = 0.02) were significantly associated with rPSC. In the multivariate analysis, Kp detection (HR: 3.83, 95% CI: 1.44–10.21, p = 0.007) and three or more bacterial species in clinical cultures (HR: 0.31, 95% CI: 0.12–0.79, p = 0.01) were independently associated with rPSC. Even when the multivariate analysis was restricted to culture data obtained within 1 year after LT, similar results were observed. Kp detection (HR: 3.47, 95% CI: 1.26–9.54, p = 0.02), the identification of three or more bacterial species in clinical cultures (HR: 0.35, 95% CI: 0.14–0.86, p = 0.02), and postoperative active UC (HR 2.52, 95% CI 1.05–6.07, p = 0.04) remained independently associated with rPSC. Bootstrap resampling analysis (1,000 iterations) showed similar results, with Kp detection (median HR: 4.10, 95% CI: 1.57–14.71), identification of three or more cultured bacterial species (median HR: 0.29, 95% CI: 0.09–0.73), and postoperative active UC (median HR: 2.43, 95% CI: 0.89–7.53).

**TABLE 2:** Univariate and multivariate Cox proportional hazards models for time to recurrence of primary sclerosing cholangitis after liver transplantation.

Variables	Univariate analysis		Multivariate analysis	
	HR 95% CI	p	HR 95%CI	p
Recipient factors				
Age <20	0.90 0.23–4.28	>0.99	​	​
Sex, Male	0.54 0.22–1.28	0.16	​	​
Mayo PSC risk score >2	1.02 0.38–2.79	0.96	​	​
HLA-DR15	0.70 0.28–1.72	0.44	​	​
Donor factors				
Age ≥45	2.22 0.91–5.42	0.08	​	​
Sex mismatch	1.02 0.44–2.36	0.97	​	​
ABO-incompatible	1.84 0.53–6.31	0.34	​	​
First degree relationship	0.99 0.41–2.38	0.98	​	​
HLA loci mismatch number ≥4	0.77 0.28–2.12	0.61	​	​
HLA-DR15	2.17 0.90–5.22	0.08	​	​
Operativeffactors				
LDLT	0.67 0.27–1.70	0.40	​	​
Operation time [min] ≥ 600	0.43 0.16–1.13	0.09	​	​
Cold ischemic time [min] ≥ 60	1.20 0.42–3.41	0.73	​	​
Postoperative factors				
Biliary tract complication	0.71 0.24–2.10	0.53	​	​
CMV infection	0.62 0.26–1.49	0.29	​	​
Immunosuppressant 1 year after LT (alone)	1.36 0.50–3.69	0.55	​	​
Immunosuppression 1 year after LT (triple)	0.93 0.38–2.30	0.87	​	​
Active UC after LT	2.53 1.08–5.88	0.03^*^	2.37 0.99–5.64	0.052
Cultures and antibiotics				
≥3 bacterial species identified in clinical cultures	0.37 0.15–0.88	0.02^*^	0.31 0.12–0.79	0.01^*^
Kp (+) in clinical cultures	2.64 1.06–6.54	0.03^*^	3.83 1.44–10.21	0.007^*^
Oral antibiotic treatment with MNZ or VCM	1.20 0.44–3.26	0.72	​	​

HR, Hazard ratio; 95%CI, 95% confidence intervals; HLA, Human leukocyte antigen; LDLT, Living donor liver transplantation; DDLT, Deceased donor liver transplantation; CMV, Cytomegalovirus; LT, Liver transplantation; UC, Ulcerative colitis; Kp, *Klebsiella pneumoniae*. *p values <0.05.

Table 3 presents an exploratory analysis of bacterial species detected in at least three patients. Only Kp showed a significant association with rPSC (p = 0.03). Supplementary Table 1 lists all other bacterial species detected in the study cohort, as well as their respective detection counts.

**TABLE 3:** Univariate Cox proportional hazards models exploratorily evaluating the association between individual bacterial species detected in postoperative clinical cultures and recurrence of primary sclerosing cholangitis after liver transplantation.

Variables	rPSC (n = 22)	Non-rPSC (n = 40)	Univariate analysis	
			HR 95%CI	p
*Klebsiella*	​	​	​	​
*Klebsiella pneumoniae* (yes/no)	7/15	3/37	2.64 1.06–6.54	0.03*
*Klebsiella* oxytoca	2/20	1/39	1.81 0.42–7.82	0.43
*Citrobacter freundii*	2/20	2/38	1.08 0.25–4.62	0.92
*Escherichia coli*	3/19	8/2	0.56 0.16–1.89	0.35
*Enterobacter cloacae*	8/14	10/30	1.02 0.42–2.46	0.97
*Enterococcus*	​	​	​	​
*Enterococcus* avium	2/20	3/37	2.14 0.48–9.53	0.31
*Enterococcus* faecium	8/14	13/27	0.95 0.40–2.26	0.90
*Enterococcus faecalis*	4/18	8/32	0.91 0.30–2.71	0.86
*Enterococcus* gallinarum	4/18	4/36	0.82 0.27–2.46	0.72
*Pseudomonas aeruginosa*	5/17	11/29	0.61 0.22–1.66	0.33
*Staphylococcus*	​	​	​	​
*Staphylococcus aureus*	5/17	7/33	1.03 0.38–2.84	0.95
*Staphylococcus* epidermidis	6/16	11/29	1.10 0.43–2.82	0.84
*Staphylococcus* haemolyticus	0/22	3/37	-	>0.99
Stenotrophomonas maltophilia	0/22	8/32	-	>0.99

PSC, primary sclerosing cholangitis; HR, hazard ratio; 95%CI, 95% confidence intervals. *p values <0.05.

### Characteristics and timing of Kp detection and recurrence-free survival

**FIGURE 3:**
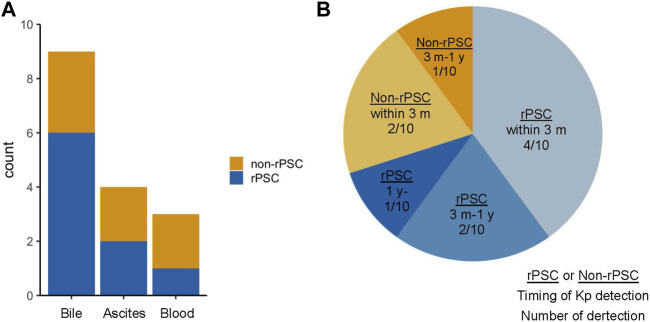
Detection profile of *Klebsiella pneumoniae* (Kp) in 10 patients after liver transplantation. **(A)** Sites of positive clinical cultures for *Kp* detection. **(B)** Timing of Kp detection following liver transplantation. RPSC: Recurrent primary sclerosing cholangitis.

**FIGURE 4:**
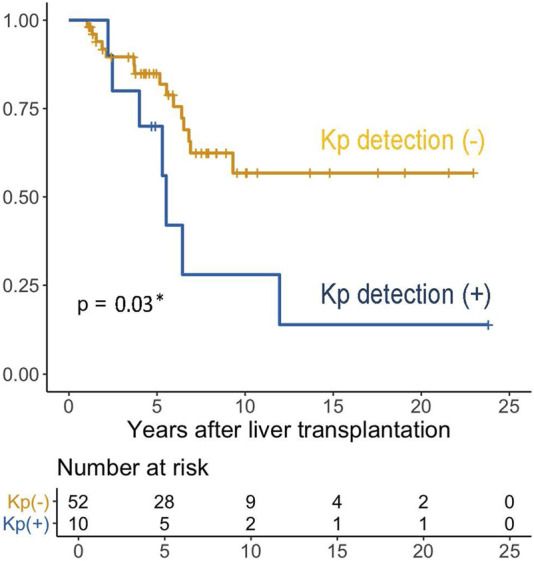
Recurrence-free survival stratified by the presence or absence of *Klebsiella pneumoniae* (Kp) detection in clinical cultures (n = 62). *p < 0.05.Kp, *Klebsiella pneumoniae*.

Figure 3 shows the types and timing of Kp detection specimens. Among the 10 patients in whom Kp was detected, Kp was found in bile in nine cases, blood in three cases, and ascitic fluid in four cases. In nine of the 10 cases, Kp was detected within 1 year post-LT, and in six cases within 3 months post-LT. Among the 10 patients with Kp detection, Kp was identified from clinical cultures obtained during suspected cholangitis episodes accompanied by symptoms such as fever or abdominal pain and/or abnormal blood test findings in four of seven rPSC patients and two of three non-rPSC patients. Figure 4 shows recurrence-free survival (RFS) stratified by Kp detection. The 5- and 10-year RFS rates were 70.0% and 28.0% in the Kp (+) group, and 84.9% and 56.7% in the Kp (−) group, respectively. RFS was significantly worse in the Kp (+) group (p = 0.03).

## Discussion

This retrospective clinical study investigated factors associated with rPSC after LT. To our knowledge, this is the first study to investigate a possible association between rPSC and Kp, which has recently been implicated in the pathogenesis of PSC. The main findings of this study are as follows: 1) rPSC was significantly associated with poorer graft survival, 2) Kp detection and lower diversity of cultured organisms were independently associated with rPSC, and 3) RFS was shorter in patients with Kp detection.

Our findings support previous studies that reported that rPSC is a major determinant of poor PGS [2–7]. Furthermore, several studies have shown that OS may not be compromised even in patients with recurrence if retransplantation is successful [5, 10, 11], and our findings were consistent with these studies. Nevertheless, some patients experience repeated episodes of rPSC and undergo multiple retransplantations, indicating that rPSC remains a significant unresolved challenge.

In the present study, the recurrence rate of PSC after LT among 76 patients was 31.6%. Although surveillance imaging, including MRCP and computed tomography, and liver biopsy were not performed according to a fully standardized protocol, patients underwent repeated examinations during follow-up. During the first 5 years after discharge, the median numbers of imaging and liver biopsy examinations per patient were 2.00 (IQR, 1.00–4.00) and 4.00 (IQR, 2.00–6.75), respectively. Furthermore, the frequencies of imaging and liver biopsy examinations did not significantly differ between the rPSC and non-rPSC groups. Compared with our previous institutional report [12], this suggests an improving trend; however, it remains higher than that reported in other studies [13, 14]. This modest improvement may be partly attributable to recent advances in UC treatment, which have improved remission rates [15, 16] and contributed to better postoperative disease control. UC has long been recognized as an important factor in PSC pathogenesis [17], and in the present study, postoperative active UC was associated with rPSC in univariate analysis, a finding that is consistent with our previous report. However, it is also notable that several recurrent cases were observed without an apparent association with UC. PSC patients in Japan have a relatively low prevalence of UC [18], highlighting the need to focus on additional factors beyond UC. Genetic factors, such as HLA, have been implicated in previous studies [12, 19]; however, these factors were not identified as independent associated factors in the present analysis. Taken together, the etiology of PSC and rPSC is likely multifactorial [1, 14]. Further investigation of additional contributing factors is warranted.

In the present study, we focused on Kp. Kp is an intestinal bacterium, that has recently been implicated in PSC pathogenesis. Kp detection in clinical cultures was independently associated with rPSC. Nakamoto et al. [8] demonstrated that Kp translocates from the colon to the mesenteric lymph nodes, induces Th17 cells, and contributes to PSC pathogenesis. Based on this evidence, we assessed the detection of bacteria, including Kp, in cultures from blood, bile, and ascitic fluid obtained between LT and the diagnosis of rPSC. Since these sites are normally sterile, bacterial detection may reflect pathological conditions such as infection or bacterial translocation from the gut. Notably, no clear association was observed for other bacterial species in this exploratory analysis, suggesting that Kp may have a distinct association with rPSC. Furthermore, lower diversity of cultured organisms was also independently associated with rPSC. Although culture-based measures do not fully capture the entire microbiome, our findings are consistent with previous reports demonstrating decreased bile microbial diversity in PSC patients [20] and dysbiosis being associated with detection of *Klebsiella* species in patients with rPSC [9]. Collectively, these observations suggest a potential contribution of gut microbiota, including Kp, in the pathogenesis of PSC and rPSC.

The clinical contribution of Kp should be interpreted with caution, as it was not detected in all patients who later developed rPSC. In this study, Kp was detected in only seven of the rPSC cases (31.8%), suggesting that its involvement is limited to a subset of patients. Nevertheless, Kp detection was associated with poorer outcomes. Similar findings were also observed when the analysis was restricted to culture data obtained within 1 year after LT. Furthermore, most Kp detections were from bile cultures obtained within 3 months postoperatively, indicating its potential early marker associated with recurrence risk. From a therapeutic perspective, we also explored the potential preventive effect of perioperative antibiotics. Although Nakamoto et al. [8] reported that oral administration of metronidazole or vancomycin improved hepatic fibrosis in a PSC mouse model, no significant association was observed in our cohort, likely due to the small sample size and limited exposure to oral antibiotics. More recently, bacteriophage therapy targeting Kp was proposed as a potential therapeutic strategy [21]. Further studies are warranted to investigate its clinical applicability in preventing rPSC.

Several limitations should be noted. First, this was a single-center retrospective study; therefore, selection and information biases cannot be excluded. Second, the criteria and timing for submitting culture samples were not standardized. Although similar findings were observed in analyses restricted to culture data obtained within 1 year after LT, potential differences between the rPSC and non-rPSC groups may still have influenced the findings. In addition, some bile and ascitic fluid cultures were obtained from postoperative drainage tubes, and Kp detection in these samples may partly reflect bacterial colonization rather than clinically significant infection. Third, this study does not indicate whether Kp detection is a cause or consequence of rPSC; thus, no definitive causal relationship can be established. Future studies should involve multicenter prospective cohorts with standardized specimen collection protocols and regulated antibiotic use. In addition, stool-based microbiota analysis and molecular or immunological characterization of Kp will be essential to elucidate its direct etiological relevance in rPSC.

In conclusion, this study is the first clinical investigation to suggest a possible association between Kp and rPSC. It represents an important step toward improving our understanding of rPSC and exploring future preventive strategies.

## Data Availability

Data involved in the present study is available from the corresponding author upon request. As the data is sourced from the hospital’s database, we are unable to publicly disclose it.
