# Supplementary material for: Postoperative detection of Klebsiella pneumoniae in clinical cultures and recurrent primary sclerosing cholangitis after liver transplantation: a single-center retrospective cohort study
Source: Transpl Int. 2026 Jun 22;39:16698. doi: 10.3389/ti.2026.16698 (PMC13333543; doi:10.3389/ti.2026.16698)
Supplement: Supplementary file 1 [file Table1.docx]

**Supplemental Table 1**. A comprehensive list of all bacterial species, not including the primary species of interest, detected in bile, blood, or ascitic fluid samples from the study cohort. Detection counts are presented according to the presence or absence of recurrent primary sclerosing cholangitis. RPSC, Recurrent primary sclerosing cholangitis.

|  | rPSC  (n=22) | Non-rPSC  (n=40) |  |  |  | rPSC  (n=22) | Non-rPSC  (n=40) |
| --- | --- | --- | --- | --- | --- | --- | --- |
| Achromobacter |  |  |  |  | Limosilactobacillus |  |  |
| Achromobacter xylosoxidans | 0/ 22 | 2/ 38 |  |  | Limosilactobacillus fermentum | 0/ 22 | 1/ 39 |
| Acinetobacter |  |  |  |  | Leclercia |  |  |
| Acinetobacter baumannii | 0/ 22 | 1/ 39 |  |  | Leclercia adecarboxylata | 1/ 21 | 0/ 40 |
| Acinetobacter lwoffii | 1/ 21 | 0/ 40 |  |  | Morganella |  |  |
| Aeromonas |  |  |  |  | Morganella morganii | 0/ 22 | 1/ 39 |
| Aeromonas caviae | 0/ 22 | 1/39 |  |  | Neisseria |  |  |
| Aeromonas hydrophila | 2/ 20 | 0/ 40 |  |  | Neisseria elongata | 0/ 22 | 1/ 39 |
| Bacteroides |  |  |  |  | Neisseria flavescens | 0/ 22 | 1/ 39 |
| Bacteroides fragilis | 1/ 21 | 0/ 40 |  |  | Parabacteroides |  |  |
| Chryseobacterium |  |  |  |  | Parabacteroides distasonis | 0/ 22 | 1/ 39 |
| Chryseobacterium indologenes | 1/ 21 | 0/ 40 |  |  | Proteus |  |  |
| Corynebacterium |  |  |  |  | Proteus mirabilis | 1/ 21 | 0/ 40 |
| Corynebacterium striatum | 1/ 21 | 0/ 40 |  |  | Proteus vulgaris | 1/ 21 | 0/ 40 |
| Citrobacter |  |  |  |  | Pseudomonas |  |  |
| Citrobacter braaki | 0/ 21 | 1/ 39 |  |  | Pseudomonas putida | 1/ 21 | 1/ 39 |
| Clostridium |  |  |  |  | Pseudomonas fluorescens | 1/ 21 | 0/ 40 |
| Clostridium perfringens | 0/ 22 | 1/ 39 |  |  | Raoultella |  |  |
| Delftia |  |  |  |  | Raoultella ornithinolytica | 0/ 22 | 2/ 38 |
| Delftia acidovorans | 0/ 22 | 1/ 39 |  |  | Rothia |  |  |
| Elizabethkingia |  |  |  |  | Rothia mucilaginosa | 0/ 22 | 1/ 39 |
| Elizabethkingia meningoseptica | 1/ 21 | 0/ 40 |  |  | Serratia |  |  |
| Enterococcus |  |  |  |  | Serratia liquefaciens | 1/ 21 | 0/ 40 |
| Enterococcus raffinosus | 0/ 22 | 1/ 39 |  |  | Staphylococcus |  |  |
| Enterococcus thailandicus | 1/ 21 | 0/ 40 |  |  | Staphylococcus capitis | 1/ 21 | 0/ 40 |
| Enterobacter |  |  |  |  | Staphylococcus hominis | 0/ 22 | 2/ 38 |
| Enterobacter asburiae | 0/ 22 | 1/ 39 |  |  | Staphylococcus lugdunensis | 0/ 22 | 1/ 39 |
| Helicobacter |  |  |  |  | Streptococcus |  |  |
| Helicobacter cinaedi | 0/ 22 | 1/ 39 |  |  | Streptococcus anginosus | 0/ 22 | 1/ 39 |
| Klebsiella |  |  |  |  | Streptococcus gallolyticus | 0/ 22 | 1/ 39 |
| Klebsiella aerogenes | 1/ 21 | 0/ 40 |  |  | Streptococcus pneumoniae | 1/ 21 | 0/ 40 |
| Lacticaseibacillus |  |  |  |  | Streptococcus sanguinis | 0/ 22 | 2/ 38 |
| Lacticaseibacillus paracasei | 0/ 22 | 1/ 39 |  |  |  |  |  |
|  |  |  |  |  |  |  |  |
